# Efficacy of Using Dendritic Cells in the Treatment of Prostate Cancer: A Systematic Review

**DOI:** 10.3390/ijms26104939

**Published:** 2025-05-21

**Authors:** Helen F. M. Pacheco, Jhessyka L. F. Fernandes, Fernanda C. R. Dias, Marina C. Deus, Daniele L. Ribeiro, Márcia A. Michelin, Marcos L. M. Gomes

**Affiliations:** 1 Department of Structural Biology, Federal University of Triângulo Mineiro, Rua Vigário Carlos, 100, 10 Andar, Uberaba CEP 38025-350, MG, Brazil; helenpacheco30@gmail.com (H.F.M.P.); d202111625@uftm.edu.br (J.L.F.F.); fernandaribeiro.dias@hotmail.com (F.C.R.D.); d202011256@uftm.edu.br (M.C.D.); marcia.michelin@uftm.edu.br (M.A.M.); 2 Department of Cell Biology, Histology, and Embryology, Federal University of Uberlandia, Av Maranhão, 1783, Uberlândia CEP 38405-318, MG, Brazil; daniele.ribeiro@ufu.br

**Keywords:** immunotherapy, cancer, vaccine, lymphocytes, immune response

## Abstract

(1) The primary prostate cancer treatment involves androgen deprivation therapy, with or without chemotherapy. Immunotherapy has emerged as a promising strategy against cancer due to its ability to modulate the immune system, overcome immune evasion, and stimulate the attack on tumor cells. Thus, this review urges an exploration of the underlying mechanisms to validate the efficacy and safety of dendritic cell immunotherapy for prostate cancer treatment. (2) An extensive literature search identified 45 eligible studies in PubMed, Web of Science, SCOPUS, and Embase databases. Phase I and II clinical trials and in vitro studies (PROSPERO registration number CRD42024538296) were analyzed to extract information on patient selection, vaccine preparation, treatment details, and disease progression. (3) Despite significant variability in vaccine development and treatment protocols, vaccines were shown to induce satisfactory immune responses, including T-cell activation, increased CD4 and CD8 cell populations, upregulated expression of HLA-A2 and HLA-DR, enhanced migratory capacity of dendritic cells, and elevated interferon levels. Cytokine responses, particularly involving Interleukin 10 (IL-10) and Interleukin 12 (IL-12), varied across studies. Immunotherapy demonstrated potential by eliciting positive immune responses, reducing PSA levels, and showing an acceptable safety profile. However, side effects such as erythema and fever were observed. (4) The analyzed treatments were well-tolerated, but variability in clinical responses and side effects underscores the need for further research to optimize the efficacy and safety of this therapeutic approach.

## 1. Introduction

Prostate cancer (PCa) is the second most common malignancy among men, with high incidence and mortality rates [1]. Brazil shows the highest absolute number of cases in South America in 2020 (32.8%), with a projected increase of 82.3% by 2040 [2]. Available treatment options for prostate cancer include active surveillance, prostatectomy, chemotherapy, radiotherapy, and hormonal or ablative therapies [3]. In cases of metastatic prostate cancer, androgen ablation therapy is highly effective and remains the most widely used treatment. However, castration resistance is an inevitable outcome for the majority of patients. Consequently, the incidence of metastatic castration-resistant prostate cancer (mCRPC) has increased in recent years, and the disease remains incurable despite continuous advances in treatment strategies that merely extend survival [4,5]. Each treatment option is associated with side effects, including toxicity, fatigue, hair loss, peripheral neuropathy, incontinence, erectile dysfunction, metastasis, and, ultimately, the development of resistance to the initial therapy. The discovery of new, cost-effective treatments with milder side effects and higher efficacy is urgently needed.

Immunotherapy and precision medicine have increasingly shaped oncology treatment, making new immunotherapeutic approaches part of the standard care in recent years [5]. The strategy behind cancer immunotherapy involves modulating the immune system to overcome immune evasion and stimulate the attack on tumor cells [6]. The goal is to target cancer cells through recognition by T cells or antibodies [7]. Dendritic cells (DCs) are professional promoters of immune responses against tumor antigens, with an exceptional ability to coordinate both innate and adaptive immune systems [6]. As antigen-presenting cells, DCs regulate adaptive immunity [8] by inducing the differentiation of cytotoxic effector cells or modulating immune system cells [9]. In this context, dendritic cell-based cancer immunotherapy offers clinical benefits. Studies have demonstrated a positive prognosis for prostate cancer cases with dendritic cell infiltration in tumors [10]. DC-based therapy has shown the ability to modify the tumor microenvironment into an immune-enriched state and enhance systemic immune responses as an active approach for treating cancer patients [6]. While DC-based immunotherapies have proven promising, it is crucial to note that the field of immunotherapy is rapidly evolving, and the effectiveness of these approaches may vary depending on the disease type, patient characteristics, and other factors [11,12].

Although immunotherapy holds promise in oncology, it faces specific challenges when applied to prostate cancer. Prostate cancer is often classified as a “cold tumor” due to its immunosuppressive microenvironment [13]. This is characterized by reduced infiltration of lymphocytes, decreased activation of antigen-presenting cells, and a higher prevalence of immunosuppressive cells. Additionally, prostate cancer exhibits a low mutational burden, which limits the availability of neoantigens for immune recognition [13,14,15]. Despite these challenges, immunotherapy has demonstrated some success in treating prostate cancer. Studies involving Sipuleucel-T, a dendritic cell-based vaccine and the only FDA-approved immunotherapy for prostate cancer, have shown improved survival in cases of metastatic castration-resistant prostate cancer [16].

The use of dendritic cells in prostate cancer immunotherapy represents a promising therapeutic approach, as it demonstrates the ability to activate antitumor T cells and promote immune responses, enhancing efficacy while minimizing the side effects of current therapies. Furthermore, the growing understanding of the nature and complexity of dendritic cells, with the development of targeted strategies, provides opportunities to optimize prostate cancer treatment. This systematic review aims to explore the underlying mechanisms through randomized controlled clinical trials to validate the large-scale efficacy and safety of dendritic cell immunotherapy for prostate cancer treatment.

## 2. Materials and Methods

### 2.1. Guiding Question and Eligibility Criteria

**Figure 1:**
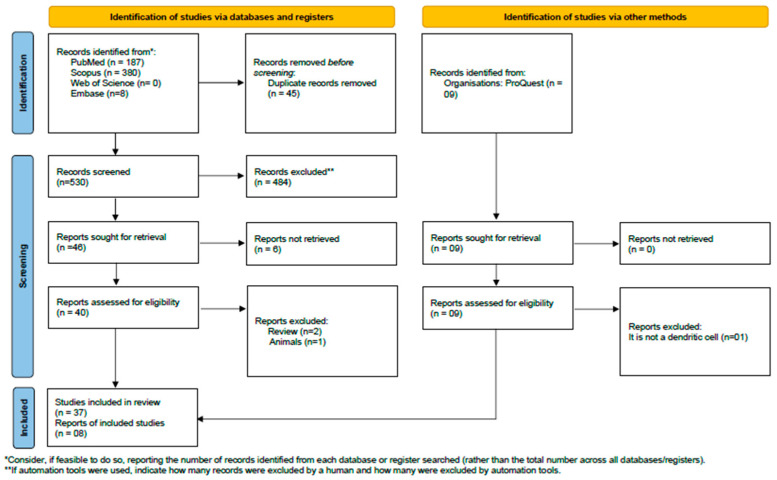
PRISMA flow diagram. Results of the bibliographic search. From [17].

This study’s guiding question was “How effective is the use of dendritic cells in combating prostate cancer?” The systematic review adhered to the PRISMA (Preferred Reporting Items for Systematic Reviews and Meta-Analyses) guidelines [17]. The PRISMA flowchart is presented in Figure 1. The methodological strategy was registered in the International Prospective Register of Systematic Reviews (PROSPERO) under registration number CRD42024538296.

The selection of studies was based on the following eligibility criteria: (1) studies evaluating dendritic cells as a method of immunotherapy, (2) studies with a methodological design including preclinical and clinical studies, (3) studies involving immunotherapy treatment for prostate cancer, and (4) dendritic cell immunotherapy treatment is monotherapy. Publications outside the scope of interest or not classified as experimental articles involving humans, as well as literature reviews, abstracts unavailable online, commentaries, notes, letters to the editor, case studies, and animal experiments, were excluded.

### 2.2. Literature Search

The bibliographic search was conducted on 28 October 2023, at 8:13 PM, across the following databases: PubMed/Medline (https://www.ncbi.nlm.nih.gov/pubmed), Scopus (https://www.scopus.com/home.uri), Web of Science (https://www.webofknowledge.com), and Embase (https://www.embase.com/landing?status=grey). The search descriptors were organized based on filters developed for two domains: (i) dendritic cells and (ii) male urogenital cancer clinical trials. Filters for PubMed/Medline were constructed following the hierarchical distribution of MeSH terms (www.ncbi.nlm.nih.gov/mesh). These filters were then adapted and applied to the Web of Science, Embase, and Scopus databases. Descriptors and keywords were combined using Boolean operators (AND/OR), and the [TIAB] algorithm was employed for non-MeSH descriptors. Studies available in English, Portuguese, and Spanish were considered for inclusion.

### 2.3. Data Extraction

Two independent reviewers (FCRD and JLFF) identified eligible studies by analyzing their titles and abstracts. Agreement between them was assessed using the Kappa test (kappa = 0.969). Any discrepancies were resolved through consultation with a third researcher (MLMG). The data recorded from each eligible study included the following: (i) publication details and study characteristics (authors, country, study type, total participants, age; informed consent form; ethics committee approval, statistical analysis; treatment criteria; androgen ablation, safety, quality of life and pain, clinical response, and follow-up treatment in responders); (ii) dendritic cell preparation (pre-stimulation, propagation, pulsation, maturation, generation, and vaccination of DCs); (iii) treatment details (experimental groups, dose, route of administration, treatment duration, treatment frequency, and systemic adjuvants); (iv) overall patient evaluation (immune responses, PSA response, acute toxicity, cytotoxicity, and cytolysis); (v) immune response evaluation (anti- and pro-inflammatory cytokines, delayed-type hypersensitivity (DTH) test, T cell stimulatory capacity, and immune response against tumor antigens); (vi) side effects; and (vii) MoDCs (MoDC phenotypes, migration, and proliferation capacity).

### 2.4. Risk of Bias (RoB)

To assess the risk of bias in the selected studies, the Cochrane Risk of Bias tool was used with a specific outcome-based approach [18]. Two team members independently evaluated the studies (FCRD and JLFF), and any disagreements were resolved by a third team member. Each study was analyzed based on the information contained within the study itself, without making assumptions. The studies were evaluated for sequence generation and allocation concealment (selection bias), participant and professional blinding (performance bias), assessor blinding (detection bias), incomplete data (attrition bias), and selective reporting (reporting bias). Additionally, the researchers of this study examined whether the studies addressed the use of informed consent, whether they underwent ethics committee approval, whether they assessed treatment safety, and whether they reported the occurrence of adverse effects. The items in the RoB tool were classified as “low risk of bias”, “high risk of bias”, or “unclear” (suggesting that the item was not reported, thus making the risk of bias uncertain).

## 3. Results

### 3.1. Description of the Main Characteristics of the Studies

The initial search yielded 575 studies: 187 from “PubMed/Medline”, 380 from Scopus, 0 from Web of Science, and 8 from Embase. Of these, 45 articles were excluded due to duplication. After reviewing titles and abstracts, 40 studies were selected for detailed examination, leaving 37 studies. The references of the eligible articles were examined, and 8 studies were added to the review, resulting in a total of 45 studies that met the inclusion criteria and were incorporated into the systematic review (Figure 1).

The included studies ranged over a 28-year period, from 1995 to 2023. From the studies included, 98% (n = 44) reported their country of origin. These studies were conducted in several countries, being the majority in the United States with 61% (n = 27), followed by the United Kingdom and Germany, with 7% (n = 3) each. The remaining studies were distributed between Switzerland and Norway, with two studies each (4.55%), and Australia, China, Denmark, Chile, the Netherlands, the Czech Republic, and Lebanon, with one study each (2.77%).

**Figure 2:**
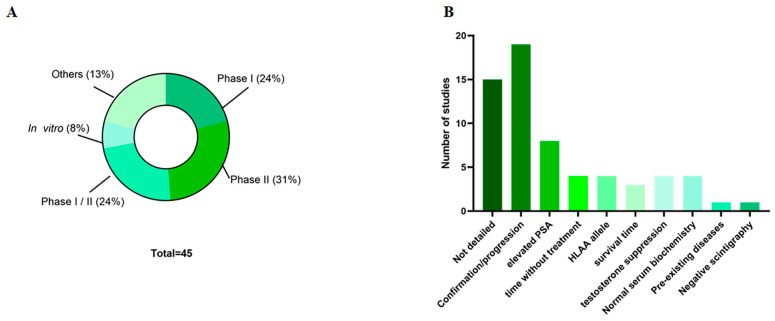
Main studied characteristics. (**A**) Types of studies; (**B**) inclusion criteria of the patients. PSA—Prostate specific antigen.

The studies were distributed between Phase I, Phase II, or a combination of both, reflecting different stages of investigation of the therapeutic strategy. The selected studies were distributed across Phase I studies (24%; n = 9), Phase II studies (31%; n = 12), or studies containing both phases (24%; n = 9), with only three of these studies being in vitro (8%) (Figure 2A). Regarding the age of the patients, 22% (n = 10) of the studies reported means and medians: the average age across all studies was 66.5 years (Table A2).

### 3.2. Patient Characteristics

Ethics committee approval for the studies was reported in only 22% (n = 10) of the studies (Table A2). Standardizing patient selection for clinical studies is important to allow comparison of results at the end of the study. Therefore, the inclusion criteria for patients in the research were detailed in 64% (n = 29) of the studies (Figure 2B). The most common criterion (38%; n = 11 studies) was that the presence and progression of cancer be confirmed histologically and that the patient’s biochemical parameters fall within normal ranges. Despite this, only 24% (n = 7) of the studies indicated elevated or altered prostate-specific antigen (PSA) levels in patients for eligibility, and 3% (n = 1) of the articles provided detailed information about the androgen ablation process.

### 3.3. Main Characteristics of Vaccine Acquisition and Preparation

Dendritic cell vaccines are primarily produced from the patient’s cells (autologous); therefore, the methods of cell collection, differentiation, and vaccine preparation are of utmost importance. Only 33% (n = 15) of the studies described how monoclonal cells were lysed: six studies (40%) used histopaque, four studies (26%) used lymphoprep, and two studies (13.3%) used leukapheresis. Only 38% (n = 17) of the studies reported acquiring dendritic cells. In 26% (n = 12) of the studies, DCs were obtained through the differentiation of peripheral blood mononuclear cells (PBMCs). The cell resuspension medium was reported in 55% (n = 25) of the articles. AIM-V and OPTIMEM media were used in 28% (n = 7 each) of the studies, 8% (n = 2) used a 5% NaCl solution, 8% (n = 2) used RPMI, and 4% (n = 1) used a specific medium for myeloid dendritic cells—Cell-Growth-DC. The primary cell propagation medium described was DCPM (Dendritic Cell Propagation Medium, n = 7), followed by AIM-V (n = 2 studies).

**Figure 3:**
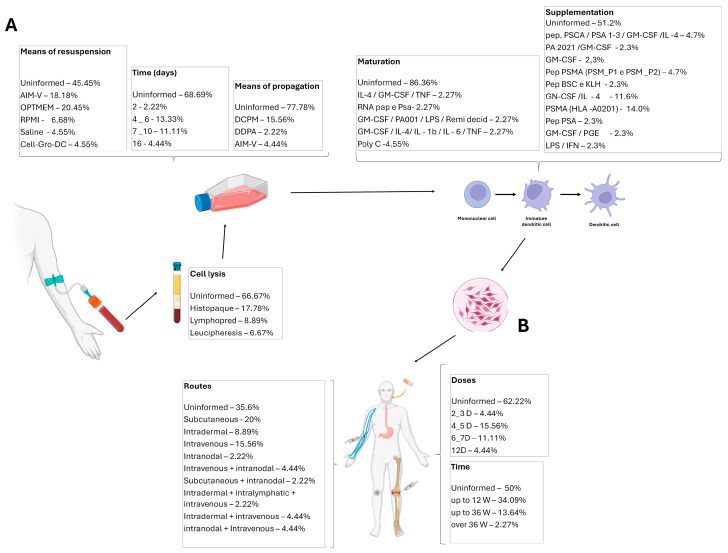
Main characteristics of the included studies. (**A**) Data on the dendritic cells’ differentiation, maturation, and harvesting. (**B**) Immunotherapy details: treatment routes, time, and doses. GM-CSF—Granulocyte-Macrophage Colony-Stimulating Factor; IL—Interleukin; LPS—Lipopolysaccharide; DCPM—Dendritic Cell Propagation Medium; TNF-α—Tumor Necrosis Factor α; IFN-γ—Interferon γ; PGE—Prostaglandin; KLH—Keyhole Limpet Hemocyanin.

The time spent in dendritic cell culture until their use in immunotherapy varies greatly, ranging from 2 to 16 days, with most studies describing times of 4 to 10 days (18%; n = 10). Dendritic cell pulsation was cited as the most commonly used specific process to enhance the efficacy of the vaccines (n = 6). Pulsation occurs with different peptides or combinations of peptides and prostate-specific membrane antigens (PSMA), androgen-sensitive human prostate adenocarcinoma cells (LNCaP), prostate acid phosphatase (PAP), or prostate stem cell antigen (PSCA). The culture of these mononuclear cells is supplemented with various substances to differentiate and mature them into fully mature dendritic cells for use in vaccines. This supplementation typically involves PSMA (HLA-A0201) (13%; n = 6), followed by Granulocyte-Macrophage Colony-Stimulating Factor (GM-CSF) and interleukin 4 (IL-4) (11%; n = 5) and smaller proportions of Prostaglandin E2 (PGE2), PSA, PSMA, or their combination (Table A3; Figure 3).

### 3.4. Main Characteristics of Treatment

Most articles provide detailed descriptions of the administered treatments (93% of studies; n = 42), such as the route of vaccine administration (n = 30). Among these, the subcutaneous route was used in 33% of the studies (n = 9), followed by intravenous (23%; n = 7), intradermal (13%; n = 4), and intranodal (3%; n = 1). Some studies combined doses or allowed for more than one of these routes. The volume administered was described in 33% (n = 15) of the studies and varied considerably (Table 1).

The treatment duration was reported in 49% (n = 22) of the studies, ranging from 2 weeks to 96 weeks, with the most common treatment duration being up to 12 weeks (66.67%; n = 14). The number of doses was described in 38% (n = 17) of the studies, varying from 2 to 14 doses, with the most common being 4 to 5 doses (43.75%; n = 7). A total of 51% (n = 23) of the articles reported treatment frequency, which was quite variable, ranging from 1 to 4 doses per week. The dose was also highly variable, ranging from 1 × 10^6^ to 10 × 10^6^. Additionally, 24% (n = 11) of the articles used systemic adjuvants in the cultures or patients during the treatment; within this percentage, 72% (n = 8) used GM-CSF. The dose of the adjuvant was described in 45% (n = 5) of the articles that used it, and its administration occurred along with the treatment using the same route (Table A3).

### 3.5. Overall Patient Evaluation

Immune response evaluation was reported in 24% (n = 11) of the studies. Among these, 36% (n = 4) indicated the assessment of T cell proliferation. In 31% (n = 14) of the studies, patient monitoring was performed, with 50% (n = 7) executing bone scintigraphy and 50% (n = 7) conducting chest X-rays. The measurement of delayed-type hypersensitivity (DTH) reactions occurred in 18% (n = 8) of the studies, carried out via the intradermal route. The assessment of T cell nonspecific response was carried out in 6% (n = 3) of the studies, while T cell proliferation assays were carried out in 4% (n = 2). The ELISA technique was utilized for supernatant analysis in 6% (n = 3) of the studies, and the Elispot assay was executed in 11% (n = 5) of the studies.

Regarding PSA response, 71% (n = 32) of the studies evaluated it, and all reported lower PSA levels in the responder group. Concerning toxicity following immunotherapy, 15% (n = 7) reported it, indicating that there was no toxicity; local reactions at injection sites were noted in 11% (n = 5); renal, hematological, and autoimmune diseases were reported in 2% (n = 1); and other grade 1 and 2 toxicities were reported in 4% (n = 2). Cytotoxicity was reported in 10% (n = 4) of the articles, and 2% (n = 1) conducted cytokine assays in microplates.

### 3.6. Immune Response

Immunotherapy aims to activate the patient’s immune system so that it can combat the established cancer. One aspect of this is the production (or lack thereof) of cytokines. TNF-α was evaluated in 4% (n = 2) of the studies, with one showing an increase and the other a weak response. IFN-γ was assessed in 22% (n = 10) of the studies, showing an increase in 60% (n = 6) of the groups, with a reduction in the others. IL-10 production decreased in 9% (n = 4) of the studies; IL-6 increased in 2% (n = 1), IL-1β increased in 2% (n = 1), and IL-12 increased in 4% (n = 2).

Another way to assess the immune response is by evaluating T cell response. CD4+ T cells were reported in 6% (n = 3) of the studies and CD8+ T cells in 11% (n = 5), with one study reporting no response and the others showing an increase. CD3, CD4, and CD8 T cells were reported in 2% (n = 1) of the studies, associated with an increased effect after immunotherapy, as described in a single article. Some studies evaluated the CD4:CD8 ratio, reported in 2% (n = 1) of the studies, showing an upward trend in the average values.

Regarding anti-CD69, anti-γ-IFN, anti-CD3, anti-CD3-PC5, anti-CD4-FITC, anti-CD8-PE, and anti-CD16-FITC antibodies, 2% (n = 1) of the articles reported on them but did not show a response. Concerning immune monitoring, 15% (n = 7) of the studies reported on it.

The delayed-type hypersensitivity (DTH) test was performed in 40% (n = 18) of the studies. Cytokine secretion was evaluated in 4% (n = 2). The regulation of costimulatory molecules was assessed in 4% (n = 2), with both showing positive results. The expression of cytokines stimulating prostate cancer cells was reported in 4% (n = 2) of the studies. Immunostimulatory cytokines were reported in 2% (n = 1) of the studies. Cytokine secretion in supernatants after various stimuli was reported in 2% (n = 1). The T cell stimulatory capacity was reported in 11% (n = 5) of the studies. Immune response against tumor antigens was reported in 26% (n = 12) of the studies.

The evaluation of regulatory T cells (Treg) was reported in 4% (n = 2) of the studies, while myeloid-derived suppressor cells (MDSC) were also analyzed in 4% (n = 2). Circulating myeloid dendritic cells (mDC), plasmacytoid dendritic cells (pDC), microRNA-155, and microRNA-223 were analyzed in 2% (n = 1) of the studies, all in the same article. The expression of HLA-A2 was analyzed in 6% (n = 3) of the studies, with positive results. The expression of HLA-DR was reported in 15% (n = 7). Gene expression profiles were described in 6% (n = 3) of the studies.

Regarding the phenotype of monocyte-derived dendritic cells (MoDCs), 6% (n = 3) of the studies reported on it. The migration capacity of MoDCs was reported in 2% (n = 1) of the studies. MoDC proliferation was reported in 2% (n = 1) of the studies. Concerning dendritic cells, the CMRF-56 antibody expression was reported in 2% (n = 1) of the studies. The proliferation of allogeneic T cells was described in 6% (n = 3) of the studies.

### 3.7. Quality Control

Regarding the quality control of immunotherapy, 15% (n = 7) of the articles reported on patient tolerance to administration, with all being well tolerated. In 11% (n = 5) of these studies, quality of life and pain were assessed, with the report indicating that disease progression was higher in individuals who did not develop an immune response.

Regarding clinical response, 12 studies (26%) addressed this, with 16% (n = 1) reporting an unknown response, 18% (n = 8) reporting follow-up of responders after treatment, and 8% (n = 4) reporting the clinical response of patients who did not complete treatment.

As for side effects, only six studies (15%) addressed these parameters, with the most common being erythema (12%; n = 5), bone pain (9%; n = 4), fever (8%; n = 4), fatigue (4%; n = 2), insomnia, and arthralgia (2%, n = 1 each). Other effects were present but with lower frequency.

### 3.8. Methodological Quality Assessment

**Figure 4:**
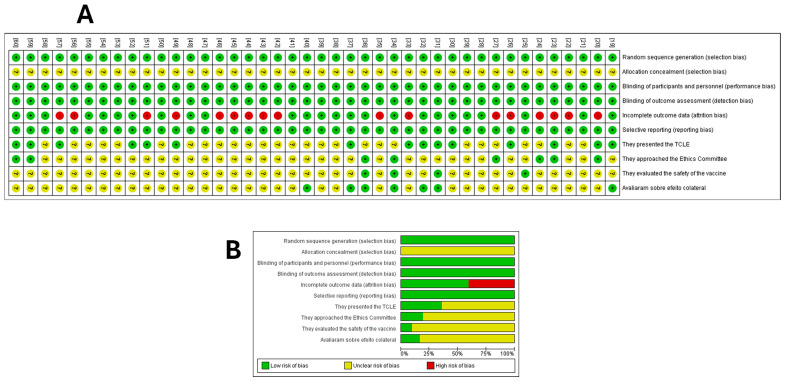
Risk of bias summary. (**A**) Methodology quality indicators for each included study (the review team’s judgments on each domain of risk of bias) at the level of the individual study. (**B**) All studies that evaluated dendritic cell immunotherapy for the treatment of prostate cancer. The Cochrane risk of bias tool was based on whether specific outcomes were marked as low risk of bias, high risk of bias, or ‘unclear risk’ (the item was not reported), resulting in an unknown risk of bias. Assessing Risk of Bias in a Randomized Trial. From [18].

The bias analysis indicated that none of the studies met all of the methodological criteria assessed (Figure 4). All studies were clear about the selection criteria, regarding sequence concealment and blinding of participants; although the studies were not always clear, we believe that, in this experimental context, blinding is inappropriate. In addition, the analyses performed also hinder bias due to the lack of blinding; therefore, we understand that this fact does not cause bias in the studies analyzed. Approximately 46% of the studies did not report attrition bias. None of the studies presented selective reporting or other factors that could potentially introduce bias in their findings.

Only 18 studies (40%) reported that participants received an informed consent term, and 9 (20%) reported that the study was approved by an ethics committee. Only four studies (8.88%) analyzed and reported on vaccine safety, while seven studies (15.55%) addressed the presence of side effects. Overall, all studies presented a methodology that was available, clear, comparable, and replicable (Figure 4).

## 4. Discussion

Androgen deprivation therapy (ADT), with or without chemotherapy, is the standard treatment for metastatic prostate cancer (mCRPC) [61]. Despite an initially favorable response to ADT, a significant proportion of patients experience relapse [62]. This relapse and progression are referred to as castration-resistant prostate cancer (CRPC), which becomes incurable and is associated with a poor prognosis [61]. In this context, interest in immune-based therapies, known as “immunotherapies”, has increased. These therapies harness patients’ immune systems to activate antigen-specific immune responses in a cascade, ultimately inducing tumor cell death [63,64].

The high incidence of prostate cancer in regions such as North America, Europe, and Australia compared to Asia and Africa [65] explains why most immunotherapy studies are conducted in countries like the United States and the United Kingdom, which account for 30 of the 45 studies included in this research. With the growing interest in immunotherapy techniques, advancements over recent decades, and the improved capacity to culture DCs, DC-based cancer vaccines have become the focus of preclinical, Phase I, II, or III clinical trials [66]. The finding underscores the significance that 92% of the studies reviewed are Phase I, Phase II, or combined Phase I/II studies.

With advancing age, immunosenescence—a decline in immune function—occurs, leading to an increased incidence of the most common cancers associated with aging [67]. For this reason, the patients included in the studies reviewed had a mean age of 66 years, although age was not one of the inclusion criteria for these studies. The fact that only 22% of the studies highlighted the approval of the research by the ethics committee is partly due to historical relationships. The importance of ethics committees, along with their implementation, acceptance, and dissemination, began to gain recognition in the last decades and is still undergoing adjustments [68]. As a result, the requirement for a clear presentation of this acceptance has also been mandated only recently. The failure to present this acceptance in the manuscript does not necessarily indicate its absence.

The primary criterion for patient inclusion in the studies reviewed was evidence of tumor progression, which could be confirmed through persistently elevated levels of PSA. PSA is the most widely used biomarker for prostate cancer [69]. As a blood-based marker, PSA is utilized across all major stages of prostate cancer management, including screening, risk stratification for recurrence, post-diagnosis surveillance, and therapy monitoring [69,70]. Despite its widespread use, histological confirmation remains the gold standard for diagnosis. However, prostate biopsy, being an invasive procedure, evaluates only a limited area of the prostate, potentially underestimating cancer aggressiveness compared to post-prostatectomy analyses [71]. While PSA testing and prostate biopsy are widely utilized techniques, emerging approaches aim to enhance the efficiency of these diagnostic tools, and additional methods can be integrated to improve their accuracy and diagnostic capabilities.

Unlike traditional vaccines, therapeutic vaccines rebuild or stimulate the body’s immune system, which previously tolerated tumor antigens. They function as modulators to transform “cold” tumors into “hot” tumors, playing a critical role in preventing metastasis and tumor recurrence [72,73]. In this context, monotherapy DCs emerge as immune cells specialized in promoting immune responses against antigens, which can include foreign pathogenic antigens or even self-tumor antigens [66]. DCs are capable of driving memory T-cell responses, but most importantly, they serve as effective initiators of naïve T-cell responses [66]. For many years, studies have focused on using DC-based vaccines against cancer to elicit and shape specific antitumor immune responses and/or enhance existing spontaneous immune responses. Since DCs are rare in circulation and tissues, the development of ex vivo culture methods has significantly increased their use in laboratory studies [66].

Most studies reviewed indicate that dendritic cells are obtained through the differentiation of peripheral blood mononuclear cells (PBMCs), largely due to the accessibility of these cells from blood samples. Mononuclear cells are isolated and differentiated using various methods. The most common method was Ficoll-Hypaque, a density-gradient centrifugation technique used to enrich mononuclear cells, improving the recovery of rare stem cells from whole blood [74]. Additionally, some studies employed leukapheresis, a procedure similar to hemodialysis, designed for safer and more efficient collection of PBMCs. This technique is widely applied in cellular immunotherapy and vaccine research [75].

Various culture media have been described for cultivating isolated mononuclear cells, with AIM-V being the most commonly used. AIM-V is a serum-free culture medium specifically designed for the proliferation and/or manipulation of T cells and dendritic cells (Fisher Scientific User Guide). The second most utilized medium is OPTIMEM, which allows for the use of reduced amounts of fetal bovine serum (FBS). OPTIMEM contains insulin, transferrin, hypoxanthine, thymidine, and trace elements that compensate for the reduction in FBS supplementation without altering growth rate or cell morphology [76]. Together, AIM-V and OPTIMEM were used in over 50% of the studies reviewed. Other media, such as RPMI and Cell-Growth-DC, were also employed but in smaller proportions.

Once mononuclear cell cultures are established, differentiation and maturation into dendritic cells (DCs) are described using various protocols. The most common method involved the use of granulocyte-macrophage colony-stimulating factor (GM-CSF) and interleukin-4 (IL-4). Many studies combined GM-CSF and IL-4 with tumor necrosis factor-alpha (TNF-α), along with IL-1β, IL-6, Prostaglandin E2 (PGE2), or monocyte-conditioned medium [77,78]. These approaches produced mature DCs with enhanced capacity to stimulate T cells [79] and improved migratory abilities [80].

Once differentiated, DC propagation predominantly occurs in the Dendritic Cell Propagation Medium (DCPM), followed by AIM-V (Fisher Scientific User Guide). Some studies employed the pulsing technique, where DCs are loaded with antigens and matured under favorable ex vivo conditions. Compared to in vivo targeting, ex vivo pulsing of DCs presents lower risks, higher efficiency, and fewer technical challenges [81]. Pulsing was performed in various ways in the studies reviewed, utilizing different peptides or combinations depending on the vaccine’s objective. Examples included prostate-specific membrane antigen (PSMA), androgen-sensitive human prostate adenocarcinoma cells (LNCaP), prostatic acid phosphatase (PAP), and prostate stem cell antigen (PSCA).

The effectiveness of immunotherapy relies on the efficient migration of DCs to secondary lymphoid organs to stimulate the immune response, as well as on the route of administration. The main administration routes were subcutaneous, followed by intravenous and intradermal, with varying vaccination schedules (weekly, biweekly, or monthly). Subcutaneous and intradermal routes enhance DC migration to lymph nodes, whereas intravenous administration results in transient pulmonary uptake before redistribution to the liver, spleen, and bone marrow [82]. Combined administration routes have been employed to improve DC migration and induce a more systemic response, as noted in 18% of the studies [11]. Systemic adjuvants were used in conjunction with antigens to enhance the antigen’s immunogenicity and provoke the desired immune response [83]. The most commonly used adjuvant in the studies was GM-CSF, which facilitates the targeting of PAP antigens to antigen-presenting cells, leading to their activation [83].

**Figure 5:**
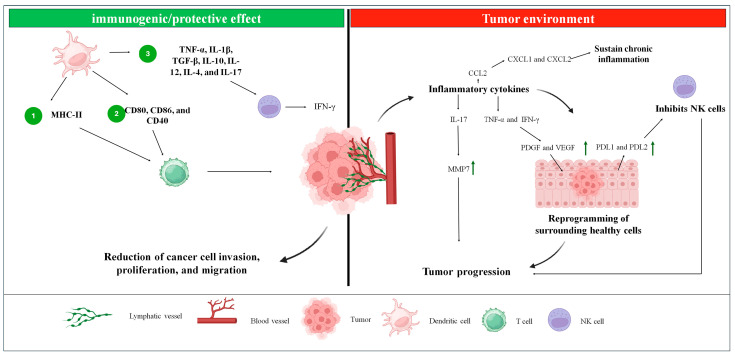
The figure represents the influence of dendritic cells in the tumor environment and how they act in controlling tumor progression. In the tumor environment, inflammatory cytokines are produced, which will either maintain chronic inflammation through the expression of CXCL1/CXCL2 or act on neighboring cells through TNF-α, IFN-γ, and IL-17 which will reprogram these cells, favoring tumor progression. The protective/immunogenic effect of dendritic cells, in turn, will stimulate NK cells to secrete interferon, which sensitizes tumor cells to T cells, which were activated by dendritic cells. Green arrow—increase in expression/production.

A satisfactory immune response is closely tied to the successful migration of dendritic cells (DCs) to lymph nodes and the production of cytokines, which reflect changes in the gene expression of DCs. These changes result in the upregulation of MHC-II, CD80, CD86, and CD40 [84,85]. DC activation can trigger CD4+ T-cell responses, including T-helper or regulatory T-cell (Treg) activation, depending on the context [86]. The DC immune response operates through three primary mechanisms: (1) MHC-II-mediated antigen presentation, which is crucial for T-cell activation; (2) upregulation of costimulatory molecules, such as CD80, CD86, and CD40, to engage and activate T cells; and (3) release of polarizing cytokines by DCs, including tumor necrosis factor-alpha (TNF-α), interleukin-1 beta (IL-1β), transforming growth factor-beta (TGF-β), and interleukin-10 (IL-10). All these pathways are essential for a productive antigen presentation [87] (Figure 5).

Post-treatment evaluations focus on disease progression, with analyses such as bone scintigraphy, chest X-rays, delayed-type hypersensitivity (DTH) testing, and PSA level measurements. Immunotherapy has been shown to reduce PSA levels by approximately 50% [34,38,41,52]. DTH testing provided the first experimental evidence of transferable immunity mediated solely by immune cells [88]. However, DTH should not be assessed in isolation but rather as a group of antigen-related responses. These responses include the tuberculin reaction, Jones–Mote reaction, contact hypersensitivity, and graft-versus-host disease. These reactions can be categorized into CD4+ and CD8+ compartments and subdivided based on T cells’ cytokine secretion patterns [89].

In this context, the immune responses involving secreted factors and cellular components within the tumor immune microenvironment of prostate cancer (PCa) can significantly influence the balance between tumor progression, elimination, and treatment response [90]. This microenvironment consists of tumor cells, immune cells, stromal cells, and the extracellular matrix. Cancer cells secrete inflammatory cytokines that reprogram surrounding healthy cells, promoting tumor growth and facilitating metastasis [91]. These cells interact with nearby tissues through the lymphatic and cardiovascular systems [92]. Various cytokines, chemokines, TNF-α, and IFN-γ play pivotal roles in PCa progression. For example, CCL2 promotes tumor progression by stimulating AKT phosphorylation, thereby enhancing invasion and metastasis. CXCL1 and CXCL2 sustain chronic inflammation and facilitate tumor development by interacting with the CXCR2 receptor [93]. Additionally, TNF-α and IFN-γ elevate levels of PDGF and VEGF via the NRF2-HIF-1α pathway and increase PDL1 and PDL2 expression in mesenchymal cells, which supports PCa growth. Polyphilin I induces p21 expression, causing cell cycle arrest at the G0/G1 phase via upregulation of IL-6. IL-8 and mTOR counter oxidative stress by inhibiting GSK-3β, while IL-17 stimulates matrix metalloproteinase 7 (MMP7) expression, driving tumor progression through epithelial-mesenchymal transition [93] (Figure 5).

ALCAR inhibits the synthesis of pro-inflammatory chemokines, TNF-α, and IFN-γ, leading to reduced invasion, proliferation, and migration of cancer cells [93]. Additionally, IL-24 suppresses cellular growth, while the IL-18-607 C/A polymorphism is associated with a reduced risk of PCa. Myeloid-derived suppressor cells (MDSCs), influenced by IL-23, contribute to resistance against androgen deprivation therapy. Furthermore, IFN-γ secreted by natural killer (NK) cells, along with high expression of IFN-γ, TNF-α, IL-5, IL-10, and macrophage inflammatory protein-1 alpha (MIP-1α), has been observed in castration-resistant prostate cancer. Among cytokines, IL-15 uniquely stimulates NK cells in the presence of PCa cells [93] (Figure 5).

Given the critical role of the immune environment in tumor progression, there is considerable interest in designing therapies that enhance immune infiltration by antigen-presenting cells (APCs) and effector T cells [94]. Consequently, assessing factors that characterize the tumor immune microenvironment is vital for understanding treatment efficacy and predicting disease progression. On this basis, an “immunoscore” has been established as a standardized metric to evaluate the tumor’s immunological context based on the density and location of CD3+ and CD8+ T cells [95]. Among the most commonly performed assessments is the analysis of T cell responses, subdivided into CD4+ and CD8+ T cells, with some studies also examining the CD4:CD8 ratio. This ratio serves as a parameter for altered immune function, often showing an increasing trend in average values. In many solid tumors, high infiltration of tumor-infiltrating lymphocytes (TILs), particularly activated CD8+ T cells, correlates with better prognoses due to their cytotoxic functions [96,97].

The use of dendritic cell (DC)-based immunotherapies is promising because these cells can regulate the type and quality of T cell responses through the production of IL-12 p70 for Th1, IL-4 for Th2, or IL-17 for Th17 responses [98,99,100]. Additionally, DCs secrete TNF, interferons, IL-10, thymic stromal lymphopoietin (TSLP), and CD40L, further influencing immune responses [66]. For these reasons, studies have evaluated levels of TNF-α, IFN, IL-1β, IL-6, IL-10, and IL-12 as key indicators of immune activation and modulation.

**Figure 6:**
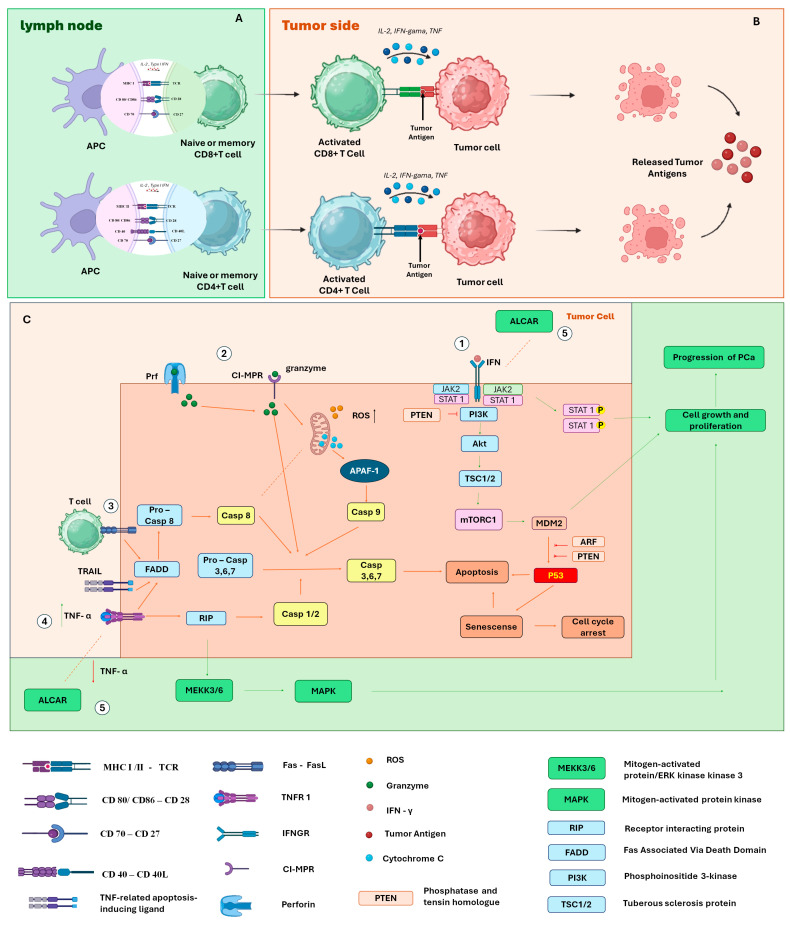
(**A**) Activation of T cells by dendritic cells in lymph nodes; (**B**) binding of T cells to tumor cells inducing apoptosis; (**C**) signaling pathways for activation of cell growth and proliferation or signaling pathways for cycle arrest or apoptosis. Pathway 1—The binding of IFN-γ to the INF-γ receptor activates the JAK-STAT signaling cascade. Dysfunctional IFN-1 signaling is directly related to determining the characteristics of the immune inflammatory environment of PCa. However, phosphatase and tensin homolog (PTEN) antagonize the pro-growth PI3K signaling pathway of PCa mediated through the ARF–MDM2–p53 axis. Pathway 2—Granzymes and perforin are packaged into cytotoxic T lymphocytes and natural killer (NK) cells. When cells bind to target cells, these proteins are released where perforin mediates the influx of granzymes through the formation of pores in the target cell membrane. The granzyme causes increased production of reactive oxygen species and release of cytochrome C by mitochondria, which activate caspase-dependent apoptotic pathways including Cas3 and Cas8. Pathway 3—Tumor recognition leads to activation of T cells and upregulation of Fas and FasL on the surface of T cells, activating the Cas8.3 cascade and ultimately apoptosis; pathway 4—TNFR1 initiates an extracellular signal by binding to “death receptors”. FAS and TRAIL are examples of these messengers that trigger the extrinsic apoptosis pathway; they bind to the TRAILR or FASR receptors, causing changes in the intracellular domain of the receptor. With these alterations, an intracellular protein FADD (Fas-associated death domain protein) is activated. It then interacts with two additional proteins, pro-caspase-8 and pro-caspase-10, which initiate the process of cell death. Pathway 5—Acetyl-L-Carnitine (ALCAR) prevents the synthesis of pro-inflammatory chemokines and TNF-α and IFN-γ, leading to a reduction in invasion, proliferation, and migration.

This modulation begins after T cells migrate to lymph nodes, where they perform short and successive initial interactions with dendritic cells (DCs), reducing their motility and inducing the expression of activation markers. Subsequently, these cells establish stable contacts with DCs, begin secreting IL-2 and IFN-γ, and finally enter a phase of rapid proliferation [101]. This process, called priming, occurs in three main stages: transient contacts, stable interaction with cytokine production, and proliferation. For complete activation to occur, recognition of the peptide–MHC complex by the T cell antigen receptor (TCR) is necessary, along with the presence of costimulatory signals provided by mature DCs [102] (Figure 6A).

The costimulatory signals required for full T cell activation involve both cytokine release and direct interactions between surface molecules. A classic example is the interaction between CD80/CD86, present on antigen-presenting cells (APCs), and CD28, expressed on T cells, which potentiates TCR-initiated signaling [103,104]. Another essential pathway is the interaction between CD40, expressed on all APCs, and CD40L, present on activated CD4 T cells. This binding promotes increased CD80/CD86 expression, stimulates IL-12 production, and supports cross-priming for exogenous antigens [105,106]. CD40 signaling on dendritic cells and other APCs plays a crucial role in inducing T cell proliferation and effector differentiation. Furthermore, this signal is essential for triggering robust responses by naïve CD8 T cells [107] (Figure 6A).

CD4 T cells help activate CD8 T cell responses largely through the delivery of CD40L to DCs, promoting their “conditioning” to induce robust effector responses by CD8 T cells [107]. In this context, CD70 expression emerges as a key component. It is a costimulatory molecule expressed by activated DCs, capable of promoting primary CD8 T cell responses even in the absence of traditional CD40 signaling. CD70 induction on DCs directly depends on contact with CD4 T cells [108], being stimulated by signals mediated by CD40L and pro-inflammatory cytokines. Once expressed, CD70 binds to the CD27 receptor on T cells, promoting their survival and the acquisition of effector functions [109,110]. Furthermore, CD70 also contributes to the production of IFN-γ by CD4 T cells, independently of IL-12 [111], reinforcing its central role in the regulation of the adaptive immune response (Figure 6A).

After activation, CD8+ T cells can form immune synapses with target cells, and their phenotype plays a central role in inducing tumor cell death. This elimination occurs through multiple mechanisms. The first involves the induction of apoptosis through the release of perforin and granzymes. Alternatively, tumor cells expressing the Fas molecule become susceptible to death when interacting with CD8+ T cells expressing the Fas ligand (FasL), also resulting in apoptosis [112]. In addition to the apoptotic pathway, CD8+ T cells can also induce tumor death through non-apoptotic mechanisms, either directly or indirectly. Recent studies have shown that these cells release cytokines such as IFN-γ and TNF-α, activating gasdermin within target cells, triggering pyroptosis—a form of lytic and inflammatory cell death [113]. Furthermore, IFN-γ can suppress the expression of glutamate–cystine transporters, compromising cystine uptake by tumor cells. This favors lipid peroxidation and leads to the induction of ferroptosis, another type of programmed cell death [114] (Figure 6A).

Although CD8+ T cells are widely recognized for their cytotoxic function, CD4+ T cells also play an essential role in tumor control. They provide critical support through the secretion of cytokines such as IFN-γ and TNF-α, which have direct antitumor activity [115]. After activation and polarization to the TH1 phenotype—in response to cytokine signaling such as IL-12 produced by dendritic cells—these CD4+ T cells begin to act more effectively [116]. In addition to cytokine support, under specific conditions, CD4+ T cells can also exert direct cytotoxicity against tumor cells, similarly to CD8+ T cells. This role has been demonstrated both in preclinical mouse models [117,118,119] and in studies with CD4+ T cells obtained from cancer patients [120] (Figure 6B).

IFN1 is crucial for an efficient antitumoral immune response, which is carried out through a variety of mechanisms, activating adaptive immune cells and facilitating the death of cytotoxic T lymphocytes [121]. The activation of the transcription factors STAT1 and STAT3 drives canonical IFN1 signaling, mediating the transcription of genes involved in stimulating and regulating both innate and adaptive immune responses [122]. Dysfunctional IFN1 signaling is directly associated with determining the characteristics of the immunoinflammatory tumor microenvironment in prostate cancer [90] (Figure 6C).

One such alteration is the loss of the PTEN protein, a lipid and protein phosphatase that antagonizes the PI3K pro-growth signaling pathway in PCa [123]. It is believed that the regulatory functions of this protein are mediated by activation of IFN1 cellular pathways [124]. Thus, PTEN is proposed to act as a tumor suppressor by regulating the PI3K–Akt–mTOR signaling network [90]. Mice deficient in both prostate-specific STAT3 and PTEN exhibit accelerated cancer progression and metastasis compared to mice deficient only in PTEN [125]. These effects are mediated through the ARF–MDM2–p53 axis, suggesting that PTEN-deficient tumors are unable to effectively activate this axis, resulting in tumor metastasis [125] (Figure 6C).

Another factor that the IFN signaling axis may impact, potentially related to tumor progression or regression, is the expression of MHC class I. The epigenetic silencing of MHC class I genes is significant in prostate cancer [126,127]. CD8+ T cells recognize antigens on the surface of cancer cells through MHC class I and, with the secretion of IFN-γ, sensitize cancer cells, inducing apoptosis via the release of perforin and granzymes [128] (Figure 6C). CD4+ T cells, which can differentiate into Tregs (regulatory T cells), also contribute to the immune system’s effectiveness in eliminating cancer cells; however, for this to occur, MHC II must be expressed on tumor cells [129].

Within the MHC, there are proteins called human leukocyte antigens (HLA). HLA-DR, mentioned in 15% of studies, is the class II MHC protein that binds to the antigen peptide and, through MHC II, is recognized by the CD4 T cell receptor (TCR). HLA-A is the protein present in MHC class I, necessary for the activation of CD8 T cells. Tumor cells also express MHC class I, and at the onset of tumor formation, this expression is positive, which enhances the recognition of tumor cells by T cells. However, there is heterogeneity in MHC phenotypes, which can evolve into negative phenotypes, thus preventing T cell responses. Positive regulation of class I HLA in tumor cells suggests metastatic regression [130].

Some studies evaluate the T cells themselves, with 6% of studies focusing on allogenic T cells, a strategy extensively explored in cellular immunotherapy interventions due to the potential to affect immune cells specifically directed against tumor cells. Autologous T cells are exposed to high levels of lactic acid, low glucose levels, and hypoxia in the tumor microenvironment, characteristics that can lead to intrinsic dysfunctions in these cells, associated with the inability to recognize tumor cells [129]. Therefore, the transfer of allogenic T cells provides an immunological advantage in immunosuppressive environments [131].

Another T cell type extensively studied is the Treg cell, evaluated in 4% of the articles. They are activated by TCR in the presence of IL-2 and co-stimulation via the CD28 pathway and are capable of inhibiting the activation and expansion of naïve conventional T cells and effector T cells, such as CD4+ and CD8+. A well-studied characteristic of Tregs is their ability to produce immunosuppressive cytokines, such as IL-10. IL-10 also contributes to the reduction of effector responses by inhibiting IL-12, decreasing IFN-γ and the Th1 pattern, and binding to antigen-presenting cells (APCs), which, through interaction with CD28 receptors, inhibits effector cell activation, promoting a tolerogenic phenotype in dendritic cells (DCs). This inactivation of dendritic cells reduces the expression of CD80 and CD86 [132]. The late expansion of Tregs induced by IL-2 after castration and immunization abolishes the initial antitumor response of CD8+ T cells [133].

One included study analyzed circulating myeloid-derived dendritic cells (mDCs) as plasmacytoid dendritic cells (pDCs). mDCs are present in peripheral organs and can recognize and present antigens. Through the expression of MHC molecules, they can recognize antigens on the surface of cells, establish immunological synapses by binding to T cell receptors, and stimulate effector T cell responses, a crucial factor in the tumor microenvironment [134]. It is believed that the overexpression of mDCs increases the production of IL-12 [135]. On the other hand, pDCs express surface markers such as toll-like receptors, which are important for pathogen recognition. When resting, pDCs have a weak capacity to stimulate T cells; however, after activation, they acquire a dendritic shape and become more effective at stimulating T cells through the expression of co-stimulatory molecules and MHC. pDCs may not only lack the ability to induce antitumoral T cells but may also induce Tregs [136,137]. These cells can transform into conventional dendritic cells upon activation and secrete type I interferon (IFN-I) [138]. Some studies also report the phagocytic capacity of pDCs. These cells appear capable of phagocytosing particulate antigens, such as apoptotic cells. Phagocytic efficiency depends on the tissue origin of activation, the antigen type, and the duration of activation. pDCs stimulate disparate T-cell responses depending on the activation stimulus, thus having the capacity to modulate the immune response of these cells [138].

The toxicity associated with immunotherapy was evaluated in only seven studies. In two of them, no toxicity was reported [43,52], while the others reported associated effects, including local reactions from the injections, renal, hematological, and autoimmune diseases, as well as other grade 1–2 toxicities [44,56,58,60]. One study reported that the observed toxicity was not related to the administered treatment [46]. Only six studies discussed side effects, with the most frequent being bone pain, fever, fatigue, insomnia, and arthralgia. Mild and transient side effects, including fever, injection site reactions, lymphadenopathy, and fatigue, were reported [139,140]. Immunotherapy safety was addressed in 7seven studies, five of which reported that it was well tolerated, showing improvements in quality of life and pain. These studies suggested that disease progression was greater in individuals who did not develop an immune response [31,33,34,49,52]. Furthermore, eight studies mentioned a long-term follow-up (up to 3 years) of patients to assess their post-treatment response and ensure its efficacy.

From studies that evaluated patients after treatment, it was observed that the response time to therapy is variable but presents a response time that ranges from 149 days [54] to 225 days [45]; however, some studies observed a response up to 370 days after treatment [52]. The studies also evaluated tumor progression and observed that patients receiving a DC vaccine have 50% less progression than conventional protocols, in addition to presenting patients with tumor remission after treatment with DC vaccine [38,60]. Another study indicates no detectable metastases [31,32] or regression of metastases [34]. Finally, one study addressed the survival time of 22 months, which is significantly higher than the overall survival of other patients [50]

## 5. Conclusions

Although there is significant variability in the methods of obtaining, differentiating, propagating, and preparing dendritic cell-based vaccines, the immunotherapy strategy for prostate cancer shows promise. Patients generally exhibit positive responses, with T-cell activation evidenced by increased CD4 and CD8 cells. An increase in interferon levels and variation in IL-10, IL-6, IL-1B, and IL-12 levels were observed, suggesting that the immune response depends on the patient’s context. Regulatory T cells, myeloid-derived suppressor cells (MDSC), dendritic cells, and positive HLA expression were also evident. These findings are essential to validate the efficacy of immunotherapy, demonstrating the safety of the vaccines, which, although they may cause some side effects, are generally mild and temporary. The variation in clinical and immunological responses indicates that more studies are needed to better standardize protocols, doses, and routes to personalize treatment based on individual immune profiles, aiming for a more robust and lasting antitumor response.

## Figures and Tables

**Table 1:** Main characteristics of obtaining and preparing the vaccine.

Cell Separation	Medium of Resupply	Propagation Medium	Time	DCs Pulsed	DC Maturation	Supplementation	Ref.
Leukapheresis	Cell Gro DC	-	6 days	Tumor cells	Poly I: C	-	[19]
-	-	-	-	-	-	-	[20]
Leukapheresis	-	-	-	-	-	-	[21]
Leukapheresis	AIM-V	-	-	-	GM-CSF/PA001 protein/rimi-ducid and LPS	-	[22]
-	-	-	-	-	IL-4; GM-CSF; rhTNF-a;	-	[23]
Leukapheresis	-	-	-	-	mRNA de PAP and PSA	-	[24]
His-topaque	AIM-V	-	7 days	-	-	IL-4 and GM-CSF	[25]
His-topaque	AIM-V	-	-	-	-	IL-4 and GM-CSF	[25]
-	AIM-V	-	16 days	-	-	rhIL-4 and rhGM-CSF	[26]
-	AIM-V	-	-	-	-	-	[27]
-	Infusion medium made from Plasma—Lyte A	-	-	-	-	LPS and IFN-ÿ	[28]
Ficoll-Hypaque	Saline solution	-	-	-	-	-	[29]
Leukapheresis	RPMI	-	-	KLH	IL-4 and GM-CSF	-	[30]
-	-	-	-	-	-	GM-CSF and (PGE2)	[31]
-	CliniMACS	-	-	-	GM LCR, IL-4 IL-1b, IL-6, TNF-a, PGE2.	HLA-A*0201 binding peptides	[32]
-	AIM-V	-	16 days	-	-	PSA peptide supplemented with rhIL-4 and rhGM-CSF	[33]
-	-	-	-	-	-	PSA peptide	[34]
-	-	-	-	-	-	-	[35]
Lymphoprep ou Histopaque	-	-	-	-	-	PSMA, peptides with specific motif of HLA-A0201 and influenza matrix peptide M158-66	[36]
-	-	-	-	-	-	-	[36]
Lymphoprep	OPTIMEM	DCPM	4–6 days	-	-	PSMA peptides with HLA-A0201 specific motif	[37]
Histopaque	OPTIMEM	DCPM	7 days	-	-	PSMA peptides with HLA-A0201 specific motif	[38]
Histopaque	OPTIMEM	-	-	-	-	PSMA peptides with HLA-A0201 specific motif	[39]
Histopaque 1077 Ficoll	OPTIMEM	-	-	-	-	PSMA peptides with HLA-A0201 specific motif	[40]
-	-	-	-	-	-	PSMA specific for HLA-A2 (PSM-P1)	[41]
Ficoll-Hypaque	-	-	4–6 days	-	-	GM-CSF and IL-4	[42]
-	RPMI	-	-	Allogeneic tumor lysates	-	GM-CSF and IL-4.	[43]
-	-	-	-	-	-	BDC and KLH peptide	[44]
-	OPTIMEM	DCPM	6 days	PSM-P1 and PSM-P2	-		[45]
-	-	DDPA	10 days	-	-	GM-CSF	[46]
-	Saline	DCPM	-	-	-	PSMA (PSM-P1 and PSM-P2)	[47]
-	AIM-V	-	-	PAP (Provenge)	-	PA2024 (PAP and GM-CSF)	[48]
-	-	AIM-V	-	-	-	-	[49]
-	-	-	2 days	PSCA, PSA1-3 and HIV	-	PSCA, PSA1-3 and HIV and IL-4 and GM-CSF	[50]
Lymphoprep	OPTIMEM	DCPM	7 days	LNCaP lysate and tetanus toxoid	-	-	[51]
-	OPTIMEM	-	-	PSMA (AL132)	-	-	[37]
Lymphoprep	OPTIMEM	DCPM	4–6 days	-	-	-	[52]
Histopaque	OPTIMEM	DCPM	7 days	-	-	-	[53]
-	-	-	-	-	-	-	[54]
-	-	-	-	-	-	-	[55]
-	-	-	-	-	-	-	[56]
-	AIM-V	AIM-V	-	-	-	-	[57]
-	-	-	-	-	-	-	[58]
Ficoll	RPMI	-	-	-	-	-	[59]
-	Cell-Gro DC	-	5 days	-	Poly I: C soluble	-	[60]

DC—Dendritic Cells; GM-CSF—Granulocyte-Macrophage Colony-Stimulating Factor; IL—Interleukin; PGE2—Prostaglandin E2; LPS—Lipopolysaccharide; KLH—Keyhole Limpet Hemocyanin; DCPM—Dendritic Cell Propagation Medium; BDC—Human Blood Dendritic Cells; PSMA—Prostate-specific Membrane Antigens; PSCA—Prostate Stem Cell Antigen; HIV—Human Immunodeficiency Virus.

## Data Availability

The data generated or analyzed during this study are provided in this review and Appendix A.

## Abbreviations

The following abbreviations are used in this manuscript:

DC	Dendritic Cells
mDC	Myeloid Dendritic Cells
pDC	Plasmacytoid Dendritic Cells
mCRPC	Metastatic Castration-Resistant Prostate Cancer
PCa	Prostate Cancer
PSA	Prostate-specific Antigen
PBMC	Peripheral Blood Mononuclear Cells
DCPM	Dendritic Cell Propagation Medium
PSMA	Prostate-specific Membrane Antigens
LNCaP	Human Prostate Adenocarcinoma Cells
PAP	Prostate Acid Phosphatase
PSCA	Prostate Stem Cell Antigen
GM-CSF	Granulocyte-Macrophage Colony-Stimulating Factor
PGE2	Prostaglandin E2
MDSC	Myeloid-derived Suppressor Cells
CRPC	Castration-resistant Prostate Cancer
DTH	Delayed-type Hypersensitivity
IFN-γ	Interferon γ
TNF-α	Tumor Necrosis Factor α
MIP-1α	Macrophage Inflammatory Protein-1 alpha
APC	Antigen Presenting Cells

## Appendix A

**Table A1:** Article’s search results.

Database	Descriptors	Items Found	Time	Date
Pubmed	Dendritic Cells	104,275	20:13:10	28 October 2023
	Male urogenital cancer clinical trial	21,023	20:12:43	28 October 2023
	#1 and #2	187	20:13:37	28 October 2023
Scopus	Dendritic Cells	785,090	21:16:10	28 October 2023
	Male urogenital cancer clinical trial	9314	21:16:40	28 October 2023
	#1 and #2	380	21:16:58	28 October 2023
Web Of Science	TS = Dendritic Cells	170,576	21:17:30	28 October 2023
	TS = male urogenital cancer clinical trial	14	21:17:06	28 October 2023
	#1 and #2	0	21:17:54	28 October 2023
Embase	Dendritic Cells	139,497	21:14:39	28 October 2023
	Male urogenital cancer clinical trial	2103	21:15:23	28 October 2023
	#1 and #2	8	21:15:35	28 October 2023

**Table A2:** Description of the main characteristics of the evaluated studies.

Country	Type of Study	Participants	Participants per Group	Age	Inclusion Criteria	Ethics (Humans)	Ethics (Animals)	Year	Ref.
Czech Republic	Phase I/II	25	-	73	Prostatic adenocarcinoma and progression of serum PSA levels and/or radiographic progression after failure of second-line hormonal manipulation in widespread metastatic disease. Adequate hematologic, hepatic, and renal function and negative results.	-	-	2015	[19]
The Netherlands	Phase II	44	-		Histologically confirmed adenocarcinoma of the prostate. Have not received any immunotherapy, docetaxel, abazitaxel, or treatment with the RANKL inhibitor denosumab. Absence of active autoimmune disease, absence of an active viral infection or allergy to shellfish or significant laboratory abnormalities.	Y	Y	2019	[20]
Norway	Phase I	18	-	-	mCRPC with metastases and an Eastern Cooperative Oncology Group (ECOG) performance status of 0–1, adequate organ function, no known hypersensitivity to vaccines or components of the cell therapy, and no contraindications to surgery.	-	-	2023	[21]
United States of America	Phase I	-	-	72	Patients were required to have progressive mCRPC and exposure to no more than one prior chemotherapy, biologic, or combination treatment regimen for mCRPC. Visceral metastasis and narcotics for pain were allowed. Castrate testosterone level ÿ50 ng/dL.	-	-	2017	[22]
Chile	Phase I	20			Histologically confirmed PC; progressive disease during combined ADT and androgen blockade (AB). Withdrawal of other oncological treatments for at least 4 weeks. Exclusion criteria included steroid therapy and second oncological disease.	Y	Y	2013	[23]
Denmark	Phase II	43		70	They have histologically confirmed metastatic adenocarcinoma of the prostate progressing despite treatment with surgical or medical castration. Exclusion criteria were a positive serological status for hepatitis B, C, and/or HIV; use of immunosuppressive drugs (including prednisolone); and the presence of severe cardiac, pulmonary, and/or autoimmune diseases.		Y	2017	[24]
United States of America	In vitro study	14			Aggressive tumor characteristics.	Y	-	2000	[25]
United States of America	In vitro study	-	-	-	-	-	Y	2001	[25]
United States of America	Phase I	16	3-average dose and 6-maximum dose	-	Patients were required to be free from the toxic effects of prior therapies and not to have received any chemotherapy, radiotherapy, or immunotherapy for at least 6 weeks prior to study entry. In patients treated with hormonal therapy, evidence of appropriate testosterone suppression was obtained before study entry.	Y	-	2002	[26]
United States of America	Phase I	12	-	-	Histologically proven adenocarcinoma of the prostate that progressed despite primary hormonal therapy; negative serologic tests for HIV, human T-cell lymphotropic virus type I, hepatitis B, and hepatitis C; adequate hematologic parameters, with total bilirubin equal to or less than two times the upper limit of normal; and aspartate aminotransferase and alanine aminotransferase equal to or less than five times the upper limit of normal.	-	Y	2000	[27]
United States of America	Clinical study	18	-	-	-	Y	-	2017	[28]
United States of America	Clinic trial (pilot)	21	6	-	They were required to have histologically documented prostate cancer with recurrent or metastatic disease measurable by an abnormal and/or rising serum prostate-specific Ag level and detectable serum PAP levels, and no hormonal manipulation or other therapies, including immunosuppressive radiation or chemotherapy, were performed during the study.	Y	-	2001	[29]
United States of America	Phase I	24	12	-	Patients who had biopsy-proven prostate cancer and progressive disease: PSA documented as elevated on 3 occasions despite testosterone levels.	Y	-	2010	[30]
United States of America	Phase I/II	27	-	49–77	Histologically confirmed prostatic adenocarcinoma. Patients after radical prostatectomy, radiotherapy, and with elevated serum PSA concentration.	Y	-	2018	[31]
Germany	Phase I	8	-	57–74	Patients with HLAA*0201 allele, life expectancy of at least 3 months, and serum PSA between 5 and 150 ng/m.	Y	-	2006	[32]
United States of America	Phase II	21	-	70	Rising PSA while receiving androgen ablation therapy and must have had a negative bone scan at the time of study entry. Karnofsky’s performance status of 60%, castrate testosterone levels, adequate hematologic function, and adequate liver function.	Y	-	2004	[33]
Germany	Clinical trial	15	12	>18	Histologically confirmed progressive prostate carcinoma expressing measurable serum PSA, HLA type A2, life expectancy of at least 3 months, at least one measurable and/or evaluable parameter, and adequate vital organ function.	-	Y	2007	[34]
United Kingdom	In vitro	-	-	-	-	-	-	2010	[35]
United States of America	Phase II	107	-	-	-	-	-	2000	[36]
Norway	Phase I /II	20	19	-	Histologically, prostate adenocarcinoma; increased PSA in three analyses. All had continued anti-hormonal treatment during the vaccination period and 3 months after therapy.	-	Y	2005	[36]
United States of America	Phase I	51	-	-	-	Y	-	1996	[37]
United States of America	Phase II	33	-	-	-	-	-	1999	[38]
United States of America	Phase II	37	-	-	-	-	-	1999b	[39]
United States of America	Phase II	28	1st group: 17; 2nd group: 11	-	-	-	-	2000	[40]
United States of America	Phase II	63	26-hormone-refractory metastatic disease; 37-suspected local recurrence	-	-	-	-	2000b	[41]
United States of America	Cohort	-	-	-	-	-	-	2004	[42]
United Kingdom	Phase I/II	20	Patients with hormone-refractory prostate cancer = 15; Patients with metastatic renal cell cancer = 5	-	-	-	-	2004	[43]
United States of America	Phase I	14	14	-	-	-	-	2015	[44]
United States of America	Phase II	40	Phase I = 12; Post-treatments (PSA 1.5) = 28	-	-	-	-	1998	[45]
Lebanon	Clinical trial	26	12	-	Documented PSA elevation on two measurements at least 2 weeks apart, a life expectancy greater than 6 months, and at least 4 weeks after surgery or radiation. Patients treated with androgen deprivation had to discontinue at least 6 weeks of all oral nonsteroidal antiandrogens and show evidence of progression.	-	-	2006	[46]
United States of America	Phase I	107	Group AI = 33; Group AII = 33; Group B = 41	AI—72 AII—62 B—66	-	-	-	1999	[47]
United States of America	Phase I/II	31	Phase I = 12; Phase II = 19	-	Histologically confirmed adenocarcinoma of the prostate with evidence of disease progression despite androgen deprivation, serum testosterone less than 50 ng/mL, and an expected survival of at least 3 months.	-	-	2000	[48]
United Kingdom	Phase II	20	A = 12; B = 8	-	Patients with histologically confirmed metastatic adenocarcinoma of the prostate, have adequate hepatic, renal, and neurological function, a life expectancy of >6 months, and a Karnofsky’s performance status of >70%.	Y	-	2005	[49]
Germany	Phase I/II	12	-	-	HLA-A*0201-positive patients with histologically proven metastatic prostate carcinoma (PCa) and tumor progression under complete androgen deprivation.	-	-	2006	[50]
United States of America	in vitro	10	-	-	Patients with histological confirmation of prostate cancer.	Y	-	1995	[51]
United States of America	in vitro	-	-	-	Patients with prostate cancer in the pre- and post-operative period of prostatectomy.	Y	-	1996	[37]
United States of America	Phase I	51	-	-	Advanced, hormone-resistant cancer.	Y	-	1997	[52]
United States of America	Phase I/II	33	-	-	Advanced prostate cancer.			1998	[53]
United States of America	Phase I/II	Phase II—107	-	-	Phase I—patients with advanced hormone-refractory prostate cancer/Phase II—clinically progressive and hormone-refractory prostate cancer (group A) and locally recurrent prostate cancer (group B).			1999	[54]
United States of America	Clinical trial—Phase II	107	-	-	Group A (individuals with hormone-refractory metastatic prostate cancer)/group B (individuals admitted with recurrence of prostate cancer after primary treatment).			1999	[55]
-	-	-	-	-	Patients with a histological diagnosis of adenocarcinoma of the prostate who had undergone prior definitive therapy for prostate cancer consisting of external beam radiotherapy and/or brachytherapy or radical prostatectomy, with or without adjuvant androgen ablation, were eligible.			2012	[56]
China	-	-	-	-	Orchiectomy or LHRH analogue augmentation.		Y	2005	[57]
Switzerland	Clinical trial Phase I/II	6	-	58–74	Patients with hormone-refractory prostate cancer documented disease progression. In the case of antiandrogen therapy, these should have been stopped at least 6 weeks before enrollment.			2006	[58]
Australia	-	-	-	-	(i) clinically localized disease that was pre-treated or part of a watchful waiting regimen; (ii) hormone-sensitive, metastatic disease, and rising PSA; and (iii) hormone-refractory metastatic disease.	Y	Y	2006	[59]
China	-	21	11	67.7	-	Y	Y	2015	[60]

PSA—Prostate-specific Antigen; LHRH—Luteinizing hormone-releasing hormone.

**Table A3:** Main features of the treatment.

Treatment	Dose	Route	Volume	Time	Duration	Frequency	Systemic Adjuvant	Dose	Ref.
DCVAC/PCa	1 × 10^7^	Subcutaneous	2.5 mL	8 weeks	12 days	1 day/week for the first two weeks, and then another 10 days/6 weeks.	-	-	[19]
A—CD1c+ mDC vaccinations. B—pDC vaccinations. C—combined CD1c+ mDC and pDC combiDC vaccinations.	A—2–5 × 10^6^ B—1–3 × 10^6^ C—3–8 × 10^6^	Intranodal	-	-	-	-	-	-	[20]
-	-	-		-	-	-	-	-	[21]
-	4, 12.5 or 40 × 10^6^ of DCs	Intradermal	200 μL	-	5–8 days	1 day/2 weeks			[22]
Mixture of autologous TAPC cells and CCH.	2 × 10^7^ TAPC and 150 mg CCH	Subcutaneous		-	-	On the 1st, 10th, 30th, and 60th.	Saline	150 mg of CCH	[23]
DCvac	5 × 10^6^ mRNA DCs	Intradermal	-	30 weeks	14 days	First 4 cycles on days 8 and 15. In the others only on day 8.	-	-	[24]
DC transfected with mRNA encoding PSA.	-	-	-	-	-	-	-	-	[25]
DC transfected with amplifietumor RNA.	-	-	-	-	-	-	-	-	[25]
DCs transfected with mRNA encoding prostate-specific antigen (PSA).	1 × 10^7^, 3 × 10^7^ or 5× 10^7^ 10^7^	Intravenous and intradermal	-	6 weeks	3 days	3 days/2 weeks	-	-	[26]
Autologous dendritic cells loaded with PAP antigen. Autologous dendritic cells exposed ex vivo to PA2024.	0.3, 0.6, and 1.0 mg	Intravenous and subcutaneous	-	-	5 days	2 days/month	GM-CSF	-	[27]
Autologous DC pulsed with TARP peptide.	-	Subcutaneous and intradermal	-	48 to 96 weeks	5 days	1 day/3 weeks	-	-	[28]
DCs loaded with recombinant PAP.	112 × 10^6^	Intravenous, intradermal, and intralinfático	I—100 mL II—4 mL	-	2 days	2 days/month	Enriched from lymphocytes.	-	[29]
DC/LNCaP; DC/ LNCaP-M1; DC/KLH; DC alone.	2–10 × 10^6^	-	-	8 weeks	4 days	-	Supplemented with 1% autologous plasma, GM-CSF (180 ng/mL).	-	[30]
Autologous DCs pulsed with the killed LNCaP cell line (DCVAC/Pca).	1 × 10^7^	Subcutaneous	5 mL	-	12 days	-	-	-	[31]
DCs loaded with a PSA-derived HLA-A*0201-restricted peptide cocktail.	1 × 10^7^	Intradermal and intravenous	2 mL ID and 50 mL EV	8 weeks	4 days	4 days/2 weeks	-	-	[32]
Dendritic cell stimulating agent Flt3 ligand.	25 g/kg	Subcutaneous	-	-	6 days	6 cycles of 28 days	-	-	[33]
IFN-g and autologous DCs pulsed with PSA peptides.	2 × 10^6^ DCs	Subcutaneous	50 mg/m^2^ of body surface	12 weeks	-	4 days/3 weeks	-	-	[34]
DC matured with lipopolysaccharide (LPS). Tumor lysate.	-	-	-	-	-	-	-	-	[35]
Autologous DCs pulsed with two HLA-A2-binding PSMA-derived peptides.	-	Intravenous	-	36 weeks	-	1 day/week	-	-	[36]
DCs transfected with mRNA from allogeneic prostate cancer cell lines (DU145, LNCaP, and PC-3).	-	Intranodal or intradermal	-	-	-	4 days/week	-	-	[36]
DC pulsed with PSMA specific for HLA-A0201.	-	Intravenous	100 mL	6–12 weeks	-	-	-	-	[37]
DC and two HLA-A2-specific PSMA peptides, PSM-P1 and -P2.	-	-	-	32 weeks	-	1 day/week	-	-	[38]
DC and two HLA-A2-specific prostate-specific membrane antigen (PSMA) peptides, PSM-P1 and -P2.	-	-	-	32 weeks	-	1 day/week	-	-	[39]
DCs pulsed with PSMA peptides.	-	-	-	6 weeks	-	1 day/week	-	-	[40]
Autologous DCs immobilized with PSM-P1 peptides.	-	-	-	-	-	1 day/week	-	-	[41]
Antigen-pulsed DC (MHC-peptide complexes).	-	-	-	-	-	-	-	-	[42]
DC pulsed with allogeneic tumor lysate (HRPC and CCR).	1–3 × 10^6^	Intranodal or intradermal	200 µL	12 weeks	-	1 day/2 weeks	-	-	[43]
CD1c (BDCA-1) + BDC.	Median of 66.95 × 10^6^	Intradermal or intravenous	-	8 weeks	-	Days 10, 28, and 56.	-	-	[44]
Autologous DC pulsed with PSM-P1 and PSM-P2.	10^6^–2 × 10^7^ DC and 2 × 10^7^ DC	-	75 g/m^2^ /day	36 weeks	-	-	GM-CSF	1000 U/ml	[45]
GM-CSF	-	-	-	-	-	-	GM-CSF	A = 125 µg/m^2^ and B 250 µg/m^2^	[46]
Autologous DC pulsed with PSMA peptides.	-	Subcutaneous	75 mcg/m^2^/day	-	-	1 day/week	GM-CSF	75 µg/m^2^/day	[47]
Pulsed DC with PAP (provenge).	100 × 10^6^	Intravenous	250 mL	24 weeks	4 days	Weeks 0, 4, and 8. A fourth infusion was administered on week 24.	-	-	[48]
-	1st dose = 3 × 10^7^ DC 2nd dose = 6 × 10^7^ DC	Intradermal	200 μL	26 weeks	-	-	-	-	[49]
Pulsed DC with PSCA.	Mean global dose 3 × 10^6^ DC	Subcutaneous	-	8 weeks	-	1 day/2 weeks	-	-	[50]
DC with LNCaP lysate.	-	-	-	-	-	-	-	-	[51]
DC with PSMA lysate.	-	-	-	-	-	-	-	-	[37]
Autologous DC pulsed with a PSM-P1 and -P2 cocktail.	1, 5, 10 ou 20 × 10^6^	Intravenous	1 g/mL	6–8 weeks	4–5 days	-	-	-	[52]
Autologous DC pulsed with a PSM-P1 and -P2 cocktail.	-	Intravenous	100 mL	6 weeks	6 days	-	GM-CSF	75 g/m^2^/day	[53]
Autologous DC pulsed with a PSM-P1 and -P2 cocktail.	Maximum acceptable dose	Intravenous		7 weeks	7 days	-	GM-CSF	-	[54]
Autologous DC pulsed with a PSM-P1 and -P2 cocktail.	-	Intravenous	100 mL	6 weeks	6 days	-	GM-CSF	-	[55]
rhGM-CSF	250 µg/m^2^ /day	Subcutaneous	-	4 weeks	14 days	-	-	-	[56]
-	-	-	-	-	-	-	-	-	[57]
DC were pulsed with the set of peptides, PSCA14–22, PAP299–307, PSMA4–12, and PSA154–163.	4 × 10^6^ DC + 10 lg/mL from each peptide	Intradermal	-	2 weeks	6 days/week	-	-	-	[58]
CD11cþCD1cþ, CD11cþCD16þ, and CD11cCD123 + BDC subsets in whole blood.	-	-	-	-	-	-	-	-	[59]
DCs pulsed with rPSMA + rSurvivin.	5 × 10^6^	Subcutaneous	-	-	5 days	3 days/15 days 2 days/month	-	-	[60]

pDC—Plasmacytoid Dendritic Cells; mDC—Myeloid Dendritic Cells; DC—Dendritic Cells; GM-CSF—Granulocyte-Macrophage Colony-Stimulating Factor; LNCaP—Human Prostate Adenocarcinoma Cells; KLH—Keyhole Limpet Hemocyanin; LPS—Lipopolysaccharide; PSMA—Prostate-specific Membrane Antigens; PSCA—Prostate Stem Cell Antigen; TAPC—T antigen presenting cell.
